# New Appliances and Things Medical

**Published:** 1905-06-03

**Authors:** 


### NEW APPLIANCES AND THINGS MEDICAL.

### GADD'S STANDARDISED GALENICALS.

(Evans, Gadd and Co., Exeter.)

We have received a number of samples of Gadd's physiologically standardised preparations. It is well recognised that in the case of those drugs containing active principles the exact chemical nature of which is not known it is difficult to be quite certain that equal volumes of a given solvent will be physiologically equally active. Indeed, there is some reason to think that, at least in the case of digitalis, plants grown in different localities do actually differ, not only in the amount of the so-called digitalin substances, but also in the activity of the latter. Under these circumstances exact dosage upon mere chemical data becomes difficult. This source of error is eliminated if we supplement chemical data with physiological; in other words, if we adopt so-called pharmacological standardisation. This of course can only be done by testing the solutions in question upon animals and finding that their activity for unit-volume is constant. The drugs that come chiefly in consideration in this regard are digitalis, aconite, strophanthus, ergot, cannabis indica, and squills, and Messrs. Gadd supply tinctures or extracts of these drugs which have been physiologically standardised, thus ensuring the possibility of an absolutely accurate dosage.

### CYLLIN.

(Jeyes' Sanitary Compounds Co., Limited, 64 Cannon Street, E.C.)

Cyllin and Cyllin (medical) are two forms of an antiseptic formerly sold as creolin by the above company. Cyllin has been the object of many experimental observations with regard to its antiseptic power and toxicity, and from these we draw the conclusion that whereas its antiseptic power is very high—actually 16 times as great as phenol—its toxicity is very low.

Cyllin is supplied in various forms, and has been used not only suitably diluted as an antiseptic and disinfectant but also for internal administration. For use as an intestinal antiseptic it can best be given in the form of palatinoids—of which we have a sample before us—each palatinoid containing 3 minims. It has also been used extensively in a special form of inhaler as an inhalant in tuberculosis of the lungs.

There can be no doubt of the efficiency of this substance as a bactericidal agent, nor of its low toxicity; we therefore think it will continue, under its new name, to be a popular antiseptic agent with the profession both for internal and external use.

